# Plant Biosystems Design for a Carbon-Neutral Bioeconomy

**DOI:** 10.34133/2020/7914051

**Published:** 2020-06-11

**Authors:** Udaya C. Kalluri, Xiaohan Yang, Stan D. Wullschleger

**Affiliations:** ^1^Biosciences Division, Oak Ridge National Laboratory, PO Box 2008, Oak Ridge, TN 37831-6422, USA; ^2^Environmental Sciences Division, Oak Ridge National Laboratory, PO Box 2008, Oak Ridge, TN 37831-6422, USA

## Abstract

Our society faces multiple daunting challenges including finding sustainable solutions towards climate change mitigation; efficient production of food, biofuels, and biomaterials; maximizing land-use efficiency; and enabling a sustainable bioeconomy. Plants can provide environmentally and economically sustainable solutions to these challenges due to their inherent capabilities for photosynthetic capture of atmospheric CO_2_, allocation of carbon to various organs and partitioning into various chemical forms, including contributions to total soil carbon. In order to enhance crop productivity and optimize chemistry simultaneously in the above- and belowground plant tissues, transformative biosystems design strategies are needed. Concerted research efforts will be required for accelerating the development of plant cultivars, genotypes, or varieties that are cooptimized in the contexts of biomass-derived fuels and/or materials aboveground and enhanced carbon sequestration belowground. Here, we briefly discuss significant knowledge gaps in our process understanding and the potential of synthetic biology in enabling advancements along the fundamental to applied research arc. Ultimately, a convergence of perspectives from academic, industrial, government, and consumer sectors will be needed to realize the potential merits of plant biosystems design for a carbon neutral bioeconomy.

Synthetic biology today is poised to tackle a variety of societal challenges. Among these are the daunting needs of addressing climate change mitigation; promoting a biology-based industry that supports production of renewable fuels, chemicals and materials; and launching a sustainable bioeconomy [1–6]. Plant systems studies under the carbon (C) capture context have primarily focused on maximizing the capacity and efficiency of photosynthesis and to a lesser extent on C allocation belowground [2]. Under the bioeconomy context, plant bioengineering efforts have mainly focused on production of biofuels and to a lesser extent on production of fibers, pharmaceuticals, and commodity precursors [5, 6]. Moreover, these limited biological C capture and bioeconomy efforts have relied on distinct deployment pathways. Developing crops dedicated to a single end use, bioenergy or biomaterials or soil sequestration, creates a challenge in competition for the limited land resource. Transformative solutions are needed in order to maximize land use efficiency and accelerate development of environmentally and economically sustainable crops that are efficient in both sequestration of C and production of economically valuable products. Here, we briefly elaborate on gaps in our process understanding and potential of synthetic biology in enabling a carbon-neutral bioeconomy, and for a defined set of objectives, identify the science and technology needs.

Plants play a central role in biogeochemical cycling of C, with photosynthetic capture of atmospheric CO_2_ in leaves, allocation of sugars to above and belowground organs, and partitioning to various chemical forms (primary and secondary metabolites and polymers), including contributions to total soil carbon. There has been a notable increase in the past decade in volume and resolution of information on biological and genetic underpinnings of root chemistry, depth, surface area, fine root production [7], root exudate and root-microbe interactions, root-shoot allometry [8], and plasticity of these traits, at finer resolutions of individual plant and root types. Root chemistry and architecture determine the efficiency of nutrient and water uptake from soil [9], root exudates, interactions with soil microbes, and formation and stability of micro- and macroaggregates [10, 11]. The optimized combinations of root chemistry and architecture can increase soil organic matter and residence time of C, resulting in an enhancement of C sequestration capability [12, 13]. It is clear that breakthroughs in technological and trait scaling approaches are affording a finer resolution into the mechanisms of C capture in plants and its transformations in soil. Greater investment in clarifying the interconnections among C transformation processes at meso- and microscale to organism and field scales, and underlying genetic controls will be critical in bridging the gaps in process understanding and improving the accuracy of biodesign and modeling efforts (Figure 1). Identification of “control knobs” in plant systems, accelerated by advanced synthetic biology approaches, will enable optimization of genotypes and cultivars that are optimally suited for both above- and belowground contributions. Recently, numerous plant genes associated with root system architecture [14] and root growth rate [15] were identified using genome-wide association studies. More recently, a root-specific gene (*PdNF-YB21*) was shown to increase both root growth and lignin content in poplar [16], which is an important bioenergy crop [17]. These genes could be coordinately expressed, under the control of root-specific promoters [18, 19] to simultaneously increase root biomass, root depth, and root lignin content of crop plants.

Biobased materials and energy derived from renewable, locally-sourced plant biomass are attractive green alternatives to petroleum-derived plastics and composites. Optimal biomass productivity under suboptimal growth conditions is a commonly desirable plant trait to produce feedstocks for biofuels, bioproducts, and biomaterials [20]. The desirable plant traits for distinct end uses are specific, for example, higher cellulose crystallinity and degree of polymerization for nanocellulose-based composites [21, 22], long-chain unbranched lignin and monomers for plastic precursors [23, 24], and a high aspect ratio and hydrophobicity of fibers for biocomposites [25]. Biomass-based green alternatives can be economically and environmentally sustainable with densification of plant traits conducive for high bioproduct yield as well as productivity on marginal lands. In order to unlock the potential of aboveground plant biomass for generating novel functionality and optimize processing and production of nanocellulose-, lignin- and biofiber-derived materials, it will be critical to expand the knowledge base along the fundamental plant sciences to applied biomaterials research arc [26, 27] (Figure 1). By leveraging the knowledge base of genetic and molecular underpinnings of stem biomass optimization knobs, plant biodesign approaches can potentially enhance product processing outcomes (e.g., accessibility, extractability, unraveling, dispersion, wetting and interfacial binding) for bio-derived monomers, polymers, fibers, and composites and also importantly render biocomponents amenable to novel functionality (acoustic and aesthetic properties) [28–31]. As applications of systems and synthetic biology approaches are showing promise in the fields of bio-derived fuels [32] and synthetic biologic materials such as adhesives and coatings [33], application of synthetic biology approaches is anticipated to facilitate advancements in bio-derived structural materials. For maximizing the potential of a C neutral bioeconomy, biosystems design strategies will need to be developed and implemented to enhance the production of biomass feedstock for bioenergy and biomaterials in the above-ground tissues (e.g., stem, leaf), while simultaneously increasing the storage of C in the belowground tissues (i.e., roots) (Figure 1).

Plant biosystems design is an emerging interdisciplinary field combining multiple research areas, such as systems biology, synthetic biology, genetic engineering, plant phenomics, and computational biology. Plant systems design involves iterative cycles of design-build-test-learn. The design step of plant biodesign requires the knowledge of biological components (e.g., composition and function of DNA, RNA, and protein sets) [34] generated by systems biology research as well as the understanding of engineering principles of synthetic biology. The build step will require a community-curated synthetic biology toolbox of theory, parts, and principles to create genetic circuits that can precisely and securely optimize biological control knobs. The test step requires the capability of genetic engineering, systems biology, and plant phenomics for generating multiomics data (genomics, transcriptomics, metabolomics, proteomics, and cellular to field-scale phenomics). The learn step relies on computational biology for integrative analysis of the multiomics data, providing guidance on the improvement of the original biological design.

The design-build-test-learn approach could be used to optimize the engineering of the genes for C capture and the genes for the production of biofuels and bioproducts to achieve appropriate tissue-specific expression. For example, the expression of lignin biosynthesis genes could be downregulated in the stem tissue to reduce the recalcitrance of biomass to biofuel conversion, while upregulated in the root tissue to increase the lignin content for long-term storage of recalcitrant C in soil. Also, the leaf tissue can be engineered with genes involved in the biosynthesis and accumulation of oils for production of biofuels and lipophilic bioproducts [35–39], genes involved in the biosynthesis of biobased commodity chemicals for production of bioplastics [40], or genes encoding pharmaceutical proteins [41, 42]. On the other hand, simultaneous expression of two genes (*PdGA20ox1* and *PtrMYB221*) driven by a developing xylem tissue-specific promoter increased both quantity and quality (i.e., reduced lignin content) in the aboveground biomass in poplar, without negative impact on root growth [43]. Plant genetic engineering is mainly performed using *Agrobacterium*-mediated transformation, which suffers a limitation in the number of genes that can be transformed each time [44]. As mentioned above, both C capture and biomass feedstock production involve various genes, which could be integrated into the desired locations (e.g., not interfering with the endogenous gene expression) in the plant genome through recombinase-mediated gene integration [45] and tissue-specific gene expression (e.g., leaf-specific gene expression for chemical, stem-specific gene expression for bioenergy feedstock, and root-specific gene expression for C storage). Innovative technological solutions will be needed to develop plant systems and evaluate performance efficacy across cellular to field scales (Figure 1). Towards co-optimizing system performance for simultaneous production of bioenergy, biomaterials, and C storage, an important scientific gap is in our understanding of crosstalk among distal plant organs. Design of synthetic cell-cell and long-distance communication systems [46] can further aid in coordinating the gene expression for optimal spatiotemporal C allocation and partitioning. Facilitation of higher throughput needed in biodesign iterations calls for wider integration of automation, robotics, artificial intelligence, and predictive modeling-based approaches into the plant science research infrastructure.

Finally, considerations of new technologies including synthetic biology and advanced biodesign should go hand in hand with risk-benefit assessments and ethical, legal, and social implications [47, 48]. Successful integration of the plant-based C biosequestration and plant-derived energy and materials concepts will be influenced by geopolitical and socioeconomic factors. Transforming the pace of progress will need both an increase in consumer demand and an infusion of intentional drivers, such as incentives, mandates, and national targets. Building enabling social ecosystems of people and partnerships across researchers, designers, farmers, industry, consumers, and policy makers will help realize the spectrum of economically and environmentally sustainable solutions [49, 50]. A convergence of perspectives from research, industrial, government, and consumer sectors will be needed to realize the merits of plant biodesign for a C neutral bioeconomy.

## Figures and Tables

**Figure 1:**
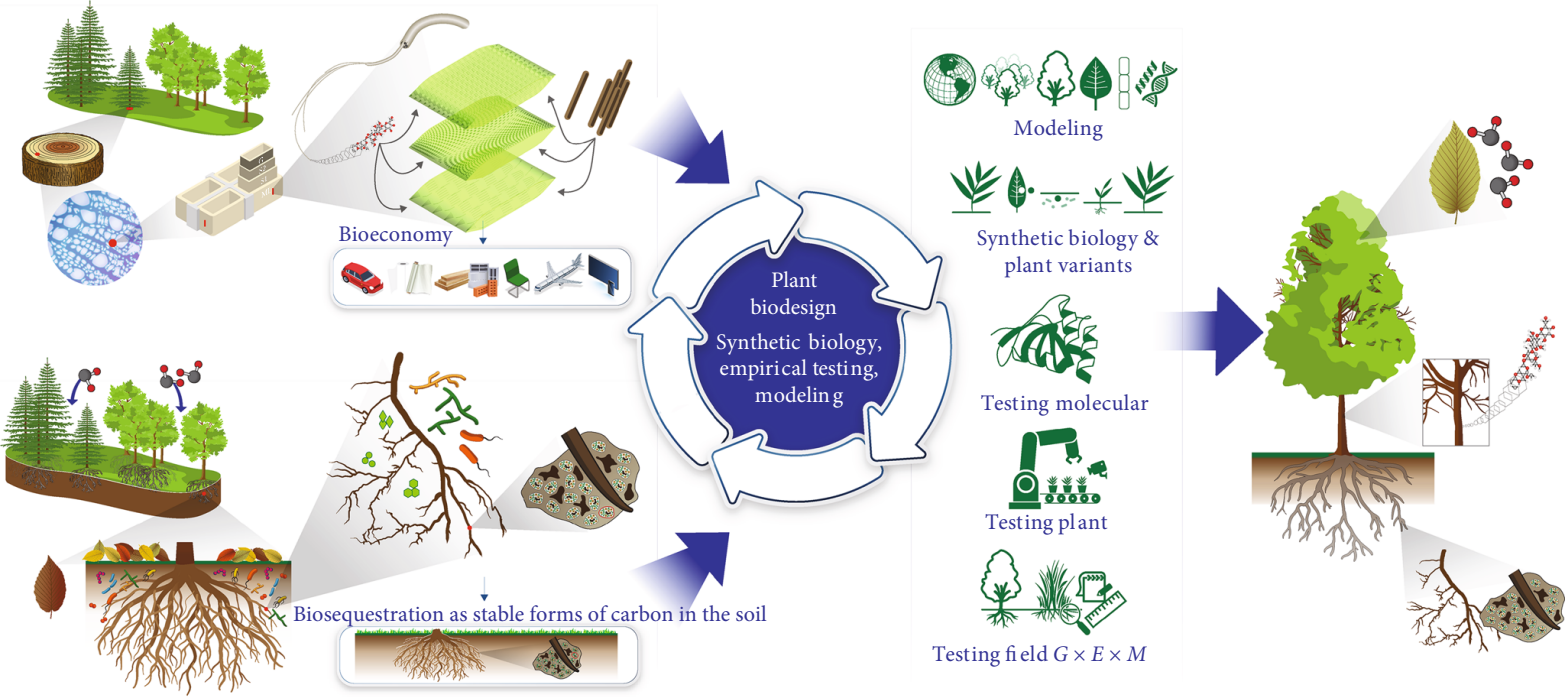
Graphical representation of the plant biosystems design for carbon-neutral bioeconomy concept. Biodesign strategies for maximizing the potential of a carbon neutral bioeconomy are needed to expand the knowledge base along the fundamental to applied research arc and to cooptimize plant chemistry and productivity. Identification of “control knobs” in plant systems, accelerated by advanced synthetic biology approaches and higher throughput in performance efficacy testing (at molecular, plant, and field levels), will enable optimization of genotypes and cultivars that are suited for both above- and belowground contributions.
